# Diseases of the Kidney

**Published:** 1903-03-07

**Authors:** 


### DISEASES OF THE KIDNEY.

**Treatment.**—Klemperer, Helen Baldwin, and Cippolina have of late investigated the causation of *oxalates* in the urine, and find that they are partly produced in the body and partly derived from the oxalic acid contained in the food. Klemperer [1] concludes that while we may redu :e the amount by careful feeding, it is not possible t › get rid of them altogether, yet it is important to prevent their precipitation. This can be to a great extent prevented by giving 30 grains of the sulphate of magnesia daily and avoiding the ingestion of lime. He allows meat fat, rice, bread, legumes, apples, coffee and alcohol, and plenty of water, but forbids milk, eggs, tea, cocoa, spinach, and cabbage.

**Granular Kidney.**—Von Noorden [2] finds that the excretion varies remarkably from day to day in this disease. It often becomes deficient as regards urea if more than an ounce is formed from the food, so too as to the uric acid, inorganic sulphates, and foreign bodies, such as lead, arsenic, and iron. Thus such patients will deal satisfactorily with 3 or 4 ounces of albuminoid food daily, and should have it except in acute exacerbations. Since they do not easily excrete more than 10 or 12 grammes of uric acid, we should not give liver, sweetbread, or other glandular meats which contain much nuclein. However, he finds that it makes absolutely no difference whether we give albumen in the form of meat, eggs, fish, or vegetables, or whether we give red or white meat such as chicken. He is opposed to the practice of giving much fluid, believing that it increases the work of the heart, and he therefore from the first restricts patients to 57 ounces daily so as to prevent the usual cardiac dilatation and failure. From numerous experiments he finds that the excretion of solids is not diminished provided that 47 ounces daily are taken besides the fluid contained in the usual solid food. To avoid any risk of the kind he allows a double allowance of water on one day in the week. While a plentiful diet is desirable, there is a danger in fattening patients. If obesity occurs this must be cautiously reduced. In acute nephritis an exclusive milk diet is desirable, but in subacute forms this cannot be long maintained. W. H. Porter [3] thinks that the causes of parenchymatous Bright's disease are excess of proteid food above the power of the organism to utilise, the presence of toxic isomeric forms of proteid due to fermentation, and the toxic products of bacteria. Alterations in the renal circulation from these and other causes lead to interstitial changes. Hence prevention of all forms of the disease depends on food regulation, so that few by-products of proteid oxidation may be poured into the kidneys. An excess of indican or uric acid indicates the strain on the organs and the first step towards disease, just as albumen from an excess diet does. Operative interference may remedy a failure of the contraction and expansion of the gland according to its normal habit, and, like nitroglycerin, give some temporary aid, but for a permanent cure we must supply the various elements of food in the amounts which the defective kidney can deal with as well as maintain the efficiency of the skin and bowels. Meat, preferably red such as beef, then eggs, milk, and lastly vegetable proteids, are in this order the best sources of supply for the necessary peptone. Nucleo-albumen and starch must be obtained from vegetable food, care being taken to avoid saccharine fermentation and interference with oxidation. In a word, anything which causes fermentation or hinders the processes of complete peptonisation or oxidation must be prevented.

[1] Practitioner, June. [2] Brit. Med. Jour., Nov. 1. [3] Med. Rec., Sept. 27.

(*To be continued.*)

